# F192. SYSTEMATIC META-ANALYSIS IDENTIFIES FIVE NOVEL ASSOCIATION LOCI FOR SCHIZOPHRENIA

**DOI:** 10.1093/schbul/sby017.723

**Published:** 2018-04-01

**Authors:** Chenxing Liu, Tetsu Kanazawa, Ye Tian, Suriati Saini, Christos Pantelis, Ian Everall, Chad Bousman

**Affiliations:** 1 University of Melbourne; 2 Osaka Medical College; 3 Institute of Psychiatry, Psychology & Neuroscience, King’s College London; 4 University of Calgary

## Abstract

**Background:**

Schizophrenia is a highly heritable psychiatric disorder. In the past 30 years, thousands of case-control and family-designed association studies have examined candidate genes for schizophrenia. To assist the field in interpreting this large volume of gene-association studies, the online SzGene database was created and included meta analyses for 287 polymorphisms at the time of its final update in 2010. However, since then more than one-thousand new gene-association studies in schizophrenia have been conducted. As such, we have conducted an updated systematic review and meta-analysis of all gene-association studies in schizophrenia published before March 2017.

**Methods:**

Studies published between January 2010 and March 2017 were identified using a customized querying strategy. Identified gene-association studies were included in the review and meta-analysis if they: 1) were case-control or family-designed studies with polymorphisms detected in schizophrenia or schizoaffective patients, 2) provide sufficient data to perform meta-analysis, and 3) were not genome-wide association studies (GWAS). Random-effects meta-analysis was performed on polymorphisms with four or more independent studies.

**Results:**

Raw data of 1711 studies included in the SzGene database were integrated with 1368 studies identified from our systematic literature search. Random-effects meta-analysis were applied to 540 polymorphisms with at least four studies and 89 of these polymorphisms were nominally associated with SZ (unadjusted p < 0.05). After bonferroni correction, 12 polymorphisms remained statistically significant, including five associations not reported in the most recent Psychiatric Genomics Consortium schizophrenia GWAS: 1) rs11098403, p = 1.45e-10, odds ratio = 0.65; 2) rs12807809, p = 7.04e-06, odds ratio = 0.91; 3) rs910694 in PDE4B: p = 1.05e-05, odds ratio = 0.77; 4) rs1801133 in MTHFR: p = 2.99e-05, odds ratio = 1.13 and 5) rs1602565: p = 7.73e-05, odds ratio = 1.11.

**Discussion:**

Our results provide a comprehensive and up-to-date review of candidate gene association studies in schizophrenia. Findings complement results from GWASs in schizophrenia but also expand the current list of candidate loci for schizophrenia.

